# Vehicular Environment Identification Based on Channel State Information and Deep Learning

**DOI:** 10.3390/s22229018

**Published:** 2022-11-21

**Authors:** Soheyb Ribouh, Rahmad Sadli, Yassin Elhillali, Atika Rivenq, Abdenour Hadid

**Affiliations:** 1 Normandie Université Rouen, LITIS (Laboratoire d’Informatique, de Traitement de l’Information et des Systèmes), Av. de l’Université le Madrillet, 76801 Saint Etienne du Rouvray, France; 2 Département d’Opto-Acousto-Électronique, DOAE, Institut d’Électronique de Microélectronique et de Nanotechnologie, IEMN, Université Polytechnique Hauts-de-France, UMR 8520, 59300 Valenciennes, France; 3 Sorbonne Center for Artificial Intelligence, Sorbonne University Abu Dhabi, Abu Dhabi P.O. Box 38044, United Arab Emirates

**Keywords:** Vehicle-To-Everything (V2X) communications, channel state information, deep learning, vehicular network, autonomous vehicle, intelligent transportation systems

## Abstract

This paper presents a novel vehicular environment identification approach based on deep learning. It consists of exploiting the vehicular wireless channel characteristics in the form of Channel State Information (CSI) in the receiver side of a connected vehicle in order to identify the environment type in which the vehicle is driving, without any need to implement specific sensors such as cameras or radars. We consider environment identification as a classification problem, and propose a new convolutional neural network (CNN) architecture to deal with it. The estimated CSI is used as the input feature to train the model. To perform the identification process, the model is targeted for implementation in an autonomous vehicle connected to a vehicular network (VN). The proposed model is extensively evaluated, showing that it can reliably recognize the surrounding environment with high accuracy (96.48%). Our model is compared to related approaches and state-of-the-art classification architectures. The experiments show that our proposed model yields favorable performance compared to all other considered methods.

## 1. Introduction

Autonomous connected vehicles have been the focus of recent research works on intelligent transportation systems (ITS), in which autonomous vehicles are anticipated to be widely used as part of the smart road vision and the next generation of transportation systems. The development of autonomous driving system aims to achieve the highest level of autonomy, at which no driver is required. When this goal is met, Vehicle-To-Everything (V2X) communications will emerge as a paramount enabler for leveraging the full potential of these vehicles. Furthermore, V2X communication is mandatory to ensure the transition from self-autonomy to full collaborative autonomy [1,2,3]. Thus, to allow connectivity between vehicles, vehicular networks (VN) should be set up in ad hoc fashion by forming Vehicle Ad Hoc Networks (VANETs) and Mobile Ad Hoc Networks (MANETs) [4].

Because V2X communication is quite important, the automotive industry is declaring its intent to deploy V2X communication technology in their future cars. Moreover, it is further supported by transportation system governments, such as the proposed mandate from the National Highway Traffic and Safety Administration (NHTSA) that suggests all vehicles have V2X capability [5]. On the other hand, the goal of deploying autonomous vehicles is to improve road safety through cooperative driving that uses the available roadway efficiently and reduces road congestion.

According to the NHTSA, most crash accidents are caused by vehicles traveling over the speed limit. Consequently, in order to provide road safety, autonomous vehicles should be aware of the speed limit and the environment. Thus identifying the type of environment in which the vehicle is driving allows the vehicle to make good a self-decision as to the correct driving speed.

**Figure 1:**
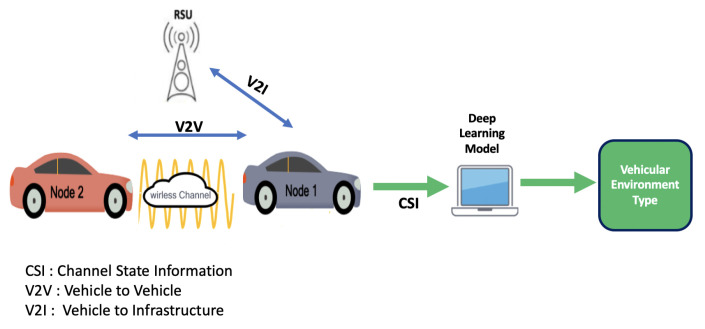
Vehicular environment identification process.

In this context, Artificial Intelligence (AI) has been established as a leading actor towards the developments of intelligent systems, enabling autonomous vehicles to make correct decisions [6,7]. Thus, in this paper we introduce a new approach towards vehicular environment identification without the need for specific sensors. The proposed method consists of using exchanged Cooperative Awareness Messages (CAM) between vehicles as well as between vehicles and infrastructure to explore channel characteristics, which are then used to recognize the vehicular environment, as shown in Figure 1.

## 2. Related Work

In the literature, many research works have focused on deep learning-based environment perception in order to make critical decisions such as vehicle speed in the context of correct decision-making for autonomous cars. In [8], the authors proposed a new method called the integrated perception approach to construct the environment. They used road information data such as the distances to surrounding lane markings provided from video images. These data were used as the input features of a neural network model in order to reach the correct driving decisions.

A highway environment identification approach has been presented in [9]. The authors used video data of a highway area recorded under various weather conditions in order to develop a vision system for recognizing the bounds of highway areas and updating the vehicle with respect to the highway driving conditions. In [10], the authors presented a new perception method for urban environments. Their approach was based on the use of video images provided from an embedded camera in a vehicle, which were then used to train a neural network in order to develop a conditional navigation model that allows for prior reception of high-level directional commands.

A vehicular urban environment perception method for autonomous vehicles was presented in [11]. The approach consists of a Global Positioning System (GPS), Radar, and Light Detection And Ranging (LiDAR)-based data fusion algorithm for reaching safe driving decisions. An environment perception approach for a self-driving vehicle in an urban area was established in [6]. In this method, the authors used a 64-beam rotating LiDAR with a specific unsupervised algorithm, then generated high-resolution maps of the surrounding environment, allowing the vehicle to enable the suitable driving parameters for its environment. The authors of [12] established an approach based on the use of data fusion in order to obtain a presentation of the environment that includes a camera, 360-degree LiDAR, and GPS/Inertial Measurement Unit (IMU) sensors deployed in a vehicle. Thus, the vehicle can make correct self-driving decisions depending on the environment in which it drives. In [13], the authors proposed an environment perception framework to enhance the environmental awareness of autonomous vehicles. This framework incorporates Voxel Region-based Convolution Neural Network (PVRCNN)-based vision features and leverages Vehicle-to-Infrastructure (V2I) communication technology. The Normal Distributions Transform (NDT) point cloud registration algorithm is used both onboard and at the roadside to obtain the position of autonomous vehicles and objects detected by the multi-sensor system at the roadside are sent back to the autonomous vehicles to improve their perception. An end-to-end machine learning model that combines control algorithms, convolutional neural networks (CNNs), and multitask (MT) learning for autonomous driving was introduced in [14]. The proposed model is able to simultaneously perform regression and classification tasks for estimating perception indicators and driving decisions, and can be used to evaluate inference efficiency and driving stability. In [15], a new approach of enhancement perception for Autonomous Driving Using Semantic and Geometric Data Fusion was presented based on low-level fusion of semantic scene information and geometry from LiDAR-based 3D point clouds. This method provides better range coverage and enables improved perception through 3D object classification and detection. In [16], the authors introduced real-time object identification, distance estimation, and instantaneous position tracking in all environmental conditions using a deep learning algorithm with no additional sensors. The proposed framework was implemented on a Raspberry Pi 4 Model B using the Raspberry Pi NoIR Camera Module V2.

Almost all of the approaches described above are essentially based on the use of specific sensors such as cameras, radars, and LIDARs. Data collection based on these sensors requires a significant amount of computing resources and power [17].

To avoid this, we propose a novel environment identification approach based on deep learning dedicated to autonomous vehicles without the need for specific sensors. For this, we exploit the shared wireless channel characteristics between vehicles communicating in vehicular networks.

Because the CSI values are the most accurate representation of wireless channel characteristics [18], we use the CSI values estimated from the packets exchanged between vehicles through Vehicle-to-Vehicle (V2V) communications as input features for our proposed convolutional neural network model. This model is able to reliably identify the surrounding environment by learning the channel characteristics (CSI) for each environment. Thus, the vehicle can set up the right automotive driving parameters (such as speed limits) corresponding to the identified environment.

The remainder of this paper is organized as follows. Section 3 describes our wireless communication model. Section 4 provides an overview of the proposed vehicular environment identification process, while Section 5 describes our tests setups and the evaluation of the performance of our proposed method. Finally, we provide our conclusions in Section 6.

## 3. System Model

To begin, we establish a wireless communication vehicular network model in which each vehicle uses a half-duplex transmitter/receiver pair to communicate with other vehicles. These vehicles exploit the wireless channel effect (characterized by CSI) on the received messages as the input features for the CNN model used to identify the vehicular environment.

**Figure 2:**
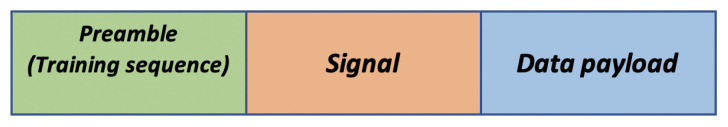
IEEE 802.11p PHY layer frame structure.

The proposed V2X network operates on the IEEE 802.11p standard. The main physical (PHY) layer of this protocol is based on the Orthogonal Frequency Division Multiplex (OFDM) waveform. The exchanged frames in the vehicular network are constituted as shown in the figure below (Figure 2).

The vehicular wireless channel is structured as a double selective fading propagation channel, which is characterized by the delay spread and the Doppler spread [18]. The base-band time-varying response of the multi-path channel is provided by

(1) $$\begin{array}{c} h(t,\tau )=\sum_{l=0}^{L-1}A_{l}\left(t,\tau \right)\delta \left(\tau -\tau_{l}\left(t\right)\right),Where\\ \;A_{l}\left(t,\tau \right)=\left|A_{l}\left(t,\tau \right)\right|\exp\left[j(2\pi f_{0}\tau_{l}\left(t\right)+\phi_{l}\left(t,\tau \right)\right] \end{array}$$

where *L* represents the number of non-zero paths, $A_{l}\left(t\right)$ represents the time-varying complex amplitudes, and $\tau_{l}\left(t\right)$ represents the time-varying path delays. Moreover, note that the phase of the complex amplitude $A_{l}\left(t\right)$ in this instance depends on the variation of Doppler shift. In addition to the time delay, the signal’s transmission over this channel may cause a Doppler shift in each path. As a result, the various delayed and frequency-shifted versions of the transmitted signal are superimposed at the receiver side [19].

We assume that the channel characteristics are static over a constant time $T_{c}$ (coherence time) [20], which is inversely proportional to the maximum Doppler shift $f_{d}$:

(2) $$T_{c}\approx \frac{0.423}{f_{d}}$$

In vehicular communication, $f_{d}$ can be expressed by the speed difference between the two communicating vehicles ΔV, as shown below:

(3) $$\begin{array}{c} f_{d}=\frac{\Delta V}{c}f_{0}\\ \;\Delta V=|V_{1}-V_{2}| \end{array}$$

where *c* and $f_{0}$ represent the celerity (speed of light) and the communication center frequency, respectively.

Depending on the coherence bandwidth ( $1/T_{c}$), when the channel’s coherence bandwidth exceeds the signal’s bandwidth, the channel exhibits flat fading. When the coherence bandwidth of the channel is smaller than the bandwidth of the signal, it is known as a frequency-selective fading channel (inter-symbol interference in the time domain).

According to the European Telecommunications Standards Institute (ETSI), the V2X scenario has a major impact on wave propagation, and thus the channel model [21]. We can consider five major vehicular environments depending on the different channel modeling characteristics of power, delay, and doppler [19,22,23]. These vehicular environment characteristics are shown in Table 1.

Because the vehicular environment is highly mobile, the transmitted messages are affected by the wireless channel. The received signal over the vehicular wireless channel can be written as

(4) Y(k)=X(k)H(k)+W(k)

where X(k) denotes the transmitted data symbols, *W* is the noise in the receiver, and H(k) denotes the wireless channel response. This channel response is characterized by the CSI. At the receiver side, a channel estimation task is mandatory; this aims to calculate the CSI, which is required in order to recover the transmitted data. Because channel estimation is quite important in V2X communications, a great deal of research work has been carried out in this field [24,25,26]. The channel estimation approaches in the literature are mainly based on observation and long training sequences (LTS). The most common channel estimation method used in industrial implementations on V2X communication boards is the LS (Least Square) estimator, thanks to its low complexity; it can be expressed as

(5) $${\hat{H}}_{LS}=\underset{H_{LS}}{min}\|Y_{t}-X_{t}.H{\|}_{2}^{2}$$

where ‖.${\|}_{2}$ is the $L_{2}$ norm, $X_{t}$ is the long training sequence vector, and $Y_{t}$ denotes the corresponding observation vector. A close optimization of the LS estimator was established in [27], as follows:

(6) $${\hat{H}}_{LS}=X_{t}^{-1}Y_{t}$$

## 4. Vehicular Environment Identification Methodology

**Figure 3:**
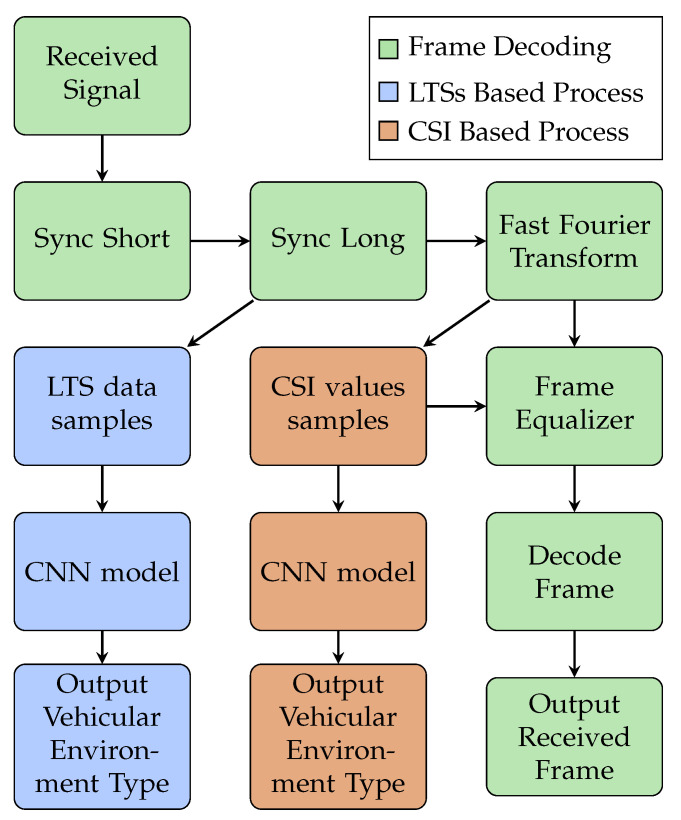
Flow chart describing vehicular environment identification process.

In this work, we consider the vehicular environment identification process as a multi-class classification problem. Thus, we propose two methods to tackle it, as shown in Figure 3. The first is based on the use of the long training sequences (LTSs) of the received frame, while the second approach is based on the calculated CSI values. Both the LTSs and the CSI values include 128 data samples, and these samples are used as the input features of the CNN model.

### 4.1. The Proposed Model

**Figure 4:**
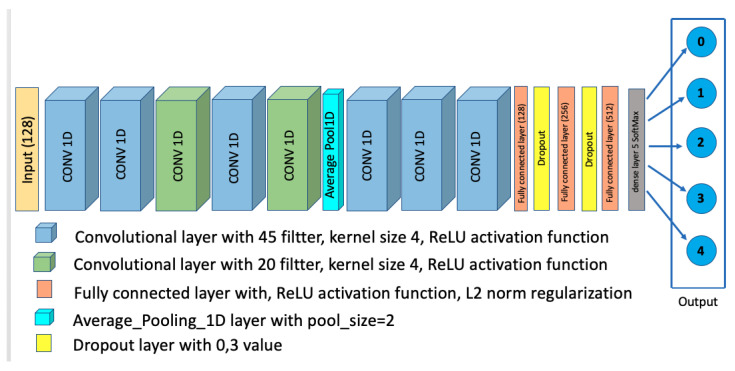
Proposed CNN Architecture.

To tackle to problem of vehicular environment identification, we propose the Convolutional Neural Network (CNN) architecture shown in Figure 4.

The proposed CNN model is constructed as follows: First, it begins with two similar one-dimensional (1D) convolutional layers; these two layers include 45 filters. Then, we have a third 1D convolutional layer, including 20 filters. This is followed by two other 1D convolutional layers that include 45 and 20 filters, respectively. The size of the filters utilized in all the previous convolutional layers is (4×1). After that, we have an average pooling layer with a pool size of 2. This average pooling layer has three 1D convolutional layers and employs 45 fillers of (4×1) kernel size. The ReLu function is used as an activation layer for all the previous convolutional layers. These layers are followed by three fully connected layers, which include 128, 256, and 512 neurons respectively, with the ReLu activation function used for these three dense layers. To reduce the overfitting effect, we add two dropout layers (with p=0,3) after the first and the second fully connected layer. Furthermore, second-norm regularization is used for all the fully connected layers [28]. Finally, the output layer is a fully connected layer in which the number of neurons is 5 (equal to the number of environment classes), with SoftMax used as the activation function.

The proposed architecture was built using the Tensorflow library [29], and we used 20 epochs and a batch size of 50 to train the model.

### 4.2. Data-Set Generation

For training, we considered 5 classes of vehicular environments: Rural LOS (Line-of-sight), Urban LOS, Urban NLOS (Non-Line-of-sight), Highway LOS, and Highway NLOS. Each environment is modeled by a wireless channel based on real-world vehicular environment measurement of the delay, gain, and Doppler frequency.

The vehicular channel characteristics of each environment can be found in Table 1. A label is assigned to each environment corresponding to the class outputs of the CNN model (Table 2).

To generate the dataset samples we employ a half-duplex V2V communication based on OFDM, which was developed using Matlab. To simulate the different vehicular environments (wireless channels models) we used the V2VChannel framework in Matlab, which is referenced in [19].

Several 802.11p packets are transmitted through the different channel models. For each environment, the packets are transmitted at a different value of the Signal-to-Noise Ratio (SNR), where the SNR range is from 15 dB to 40 dB with a step of 0.5 dB.

This process was repeated 400 times with different releases of the channel model for each environment. At each step, we computed the LTS in the received packet and the calculated CSI values, obtaining the 128 symbols (features) of both LTS and CSI associated with the specific label corresponding to each environment, as shown in Table 2.

The saved sequence features ($F_{i}$) for either CSI or LTS can be expressed as

(7) $$F_{i}=\left[\left[A\left(1\right),A\left(2\right),....,A\left(N\right)\right]\right]$$

where A(i) is the CSI or LTS sample. At the end of the process, we had 100,000 dataset samples, of which we used 80% as the training set and 20% as the validation set.

## 5. Evaluation and Results

In order to assess the validity and accuracy of the proposed model, we evaluated the system on different datasets. We generated a test set by transmitting several 802.11p packets through the different channel models (V2VChannel framework of Matlab). For each environment, the SNR range was set from 15dB to 40 dB with a step of 0.25 dB. This process was repeated 30 times with different releases of the channel model for each environment, resulting in 15,000 test sequences (LTS and CSI).

Before evaluating the proposed architecture, we trained our model using the categorical cross-entropy loss function and the Adam optimizer [30]. The training process was carried out on a machine including an NVIDIA Tesla P100 GPU.

Because both the CSI and LTS are complex numbers, we use training and test sets with three configurations depending on the input data format for each configuration. We use the magnitude for the first test-bed, the angle for the second, and a two-channel input for the third one, wherein the real part of the complex number is used for the first-channel input and the imaginary part is set for the second-channel input.

### 5.1. LTS Approach Performance Evaluation

As shown in Table 3, the two-channel configurations has high accuracy, achieving 93.42%, which is better than the magnitude and the angle configurations, which are 92.22% and 91.78% respectively.

**Figure 5:**
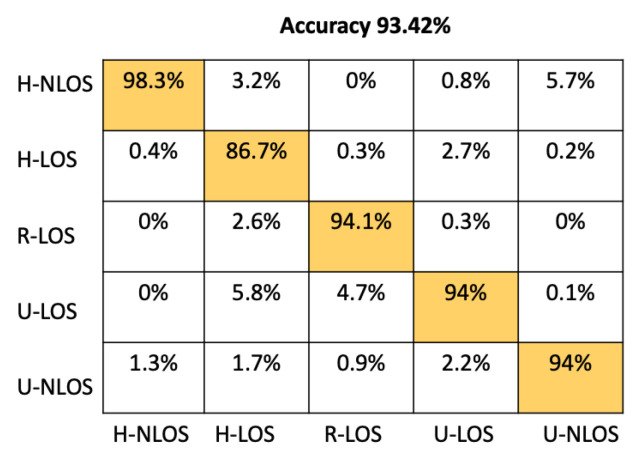
Confusion matrix based on LTS approach for the proposed CNN.

Figure 5 represents the confusion matrix of the test samples for the proposed CNN architecture using the LTS as input features within a two-channel configuration. From this confusion matrix, it can be seen that our proposed CNN model is able to reliably recognize the different vehicular environment; it correctly identifies the H-NLOS and H-LOS environments with an individual accuracy of 98.3% and 86.7%, respectively, and the R-LOS, U-LOS, and U-NLOS environments with 94% accuracy.

We compared the proposed CNN architecture to an ANN architecture containing four dense fully connected layers, including 64, 128, 256, and 512 neurons before the output layer, each of which have five neurons (equal to the number of environments to identify). Other machine learning classifier candidates used for comparison are the Random Forest classifier (RF, with 100 trees), K-Neighbors classifier (K-NN), where K was set to five neighbors, Gaussian Naive Bayes (GNB), and Support Vector Machine (SVM) with a linear kernel.

Table 4 shows the comparison between our proposed model and the approaches mentioned above based on test accuracy and environment identification prediction time. The prediction time has been determined using an NVIDIA Tesla P100 GPU. From Table 4, it is obvious that the prediction time of our proposed CNN Architecture has better performance than either SVM or K-NN, providing a prediction time of 51.33 μs. This time is comparable to the other approaches (ANN, RF, GNB) that have llowess prediction times; moreover, the accuracy of our model is significantly greater than these approaches; indeed, it has the best overall test accuracy at 93.42%.

**Figure 6:**
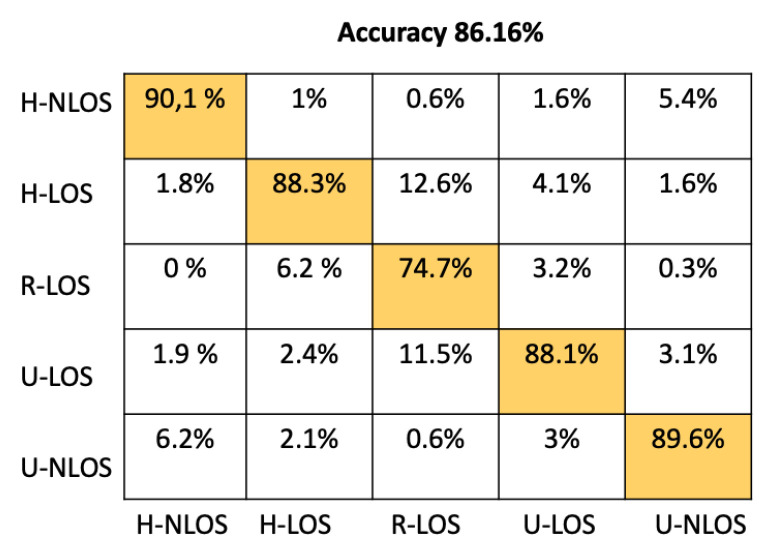
Confusion matrix for ANN based on LTS approach.

**Figure 7:**
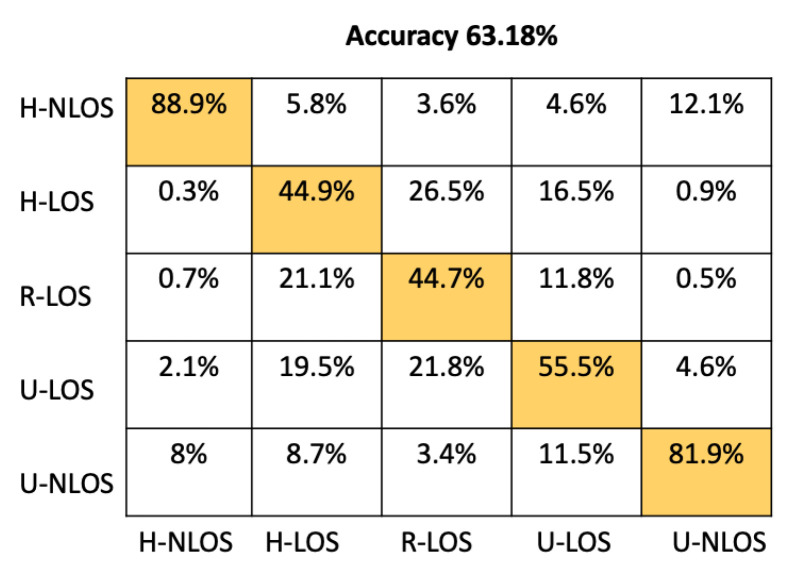
Confusion matrix for KNN based on LTS approach.

**Figure 8:**
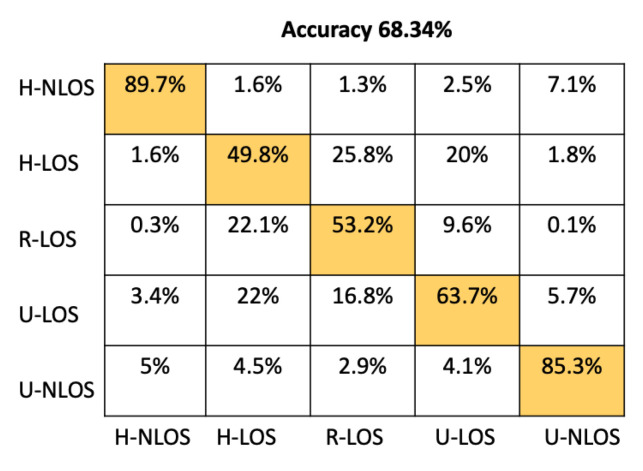
Confusion matrix for RF based on LTS approach.

**Figure 9:**
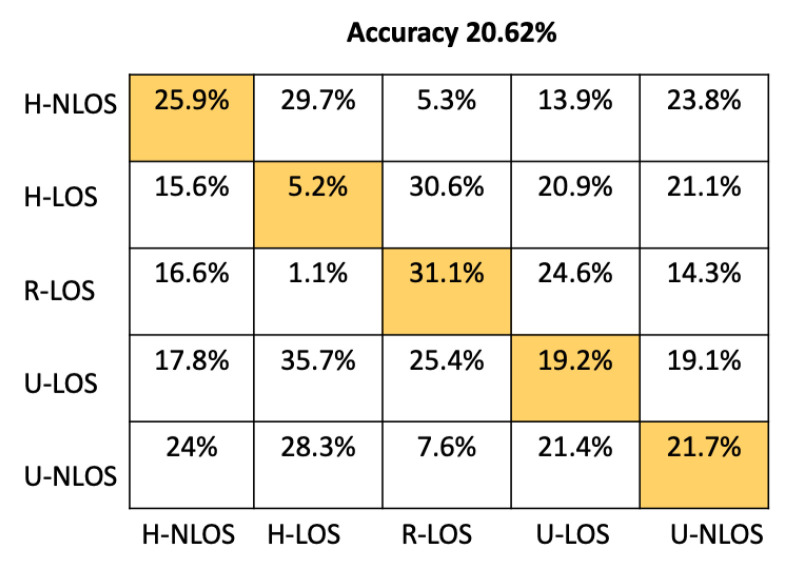
Confusion matrix for GNB based on LTS approach.

**Figure 10:**
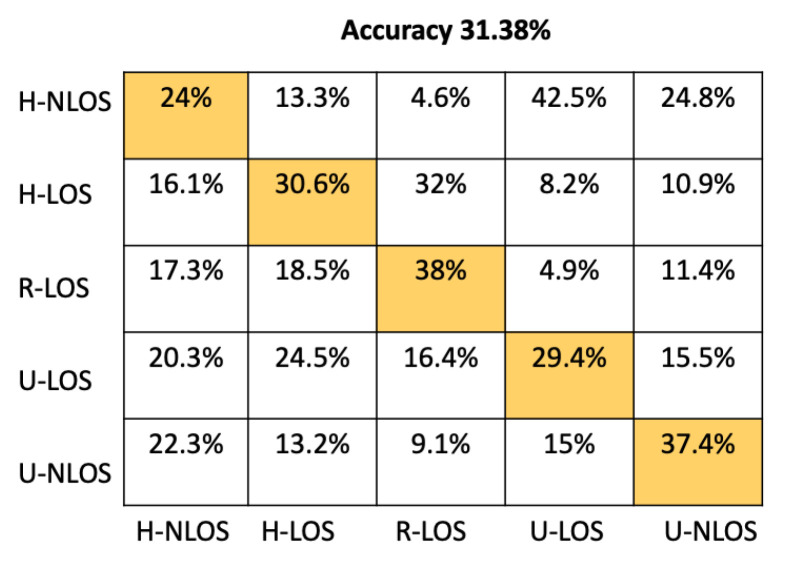
Confusion matrix for SVM based on LTS approach.

In order to provide more detail about the test accuracy classification, the confusion matrices of all the considered approaches are presented in Figure 5, Figure 6, Figure 7, Figure 8, Figure 9 and Figure 10.

It can be seen that the K-NN and RF approaches can identify H-NLOS and U-LOS environments with acceptable individual accuracy (up to 80%) and provide less than 65% of individual test accuracy for H-LOS, R-LOS, and U-LOS environments. From Figure 9 and Figure 10, it is clear that both the GNB and SVM classifiers fail to provide reliable environment identification.

### 5.2. CSI Approach Performance Evaluation

Vehicular environment identification based on the CSI approach was performed using three input feature configuration: two-channel, magnitude, and angle.

From Table 5, it is clear that that the two-channel input feature configuration provides the best performances; it has 96.48% accuracy, which is greater than the accuracy provided by the LTS approach (93.42% in two-channel configuration).

**Figure 11:**
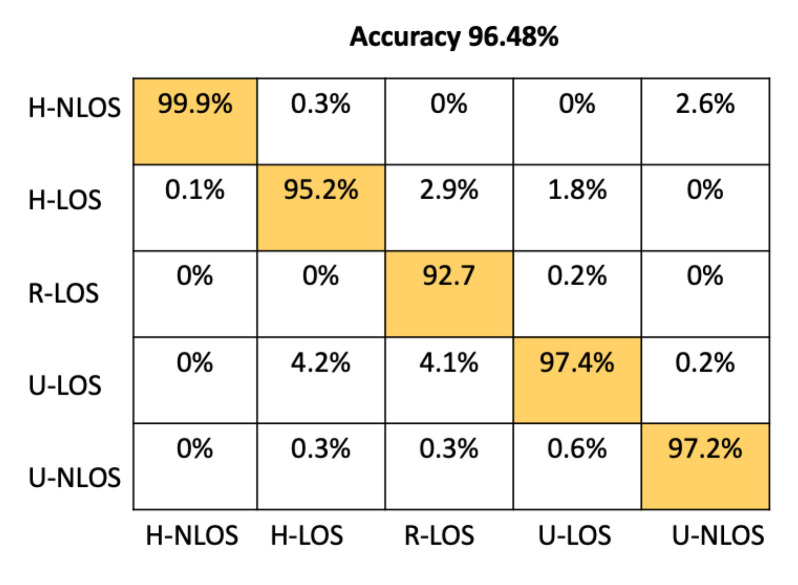
Confusion matrix for the proposed CNN based on CSI approach.

Figure 11 presents the confusion matrix of the proposed CNN architecture on the test set when using CSI values as input features for the model. The presented results are calculated taking into account a two-channel input shape, as this provides the most accurate performance. From this confusion matrix, it is clearly apparent that our proposed model can reliably identify all the vehicular environments based on CSI values, with high individual accuracy up to 92%.

Our model achieves 99.9%, 95.2%, 92.7%, 97.4%, and 97.2% for H-NLOS, H-LOS, R-LOS, U-LOS, and U-LOS environments, respectively.

The proposed CNN model based on CSI was compared to the related machine learning classifiers RF, K-NN, GBN, and SVM, as well as to an ANN architecture, in terms of test accuracy and the average time required to identify the environment (prediction time, performed using an NVIDIA Tesla P100 GPU). The ANN architecture and parameter settings of these classifiers are the same as described in Section 5.1.

From Table 6, it can be seen that our proposed CNN model has better performance than either SVM or K-NN in terms of prediction time, as it yields in 39.56 μs. While this achieved prediction time is comparable to the other approaches (ANN, RF, GNB) that have low prediction time, in term of the test accuracy our CNN model highly outperforms all the other approaches, reaching 96.48%.

According to these performance results, it is clear that the CSI approach is more accurate than the LTS approach in terms of both test accuracy and environment prediction time.

### 5.3. Comparison between Our Model and State-of-the-Art Architectures

Our proposed CNN architecture was compared against the popular state-of-the-art classification architectures ResNet50 [31], Xception [32], InceptionV3 [33], InceptionResNetV2 [34], DenseNet201 [35], and MobileNetV2 [36]. We trained these architectures on the same training datasets. Then, we evaluated their classification performances on the test set. Prior to this process, we updated the input shape of the input layer to fit our data inputs and updated the output layer size to five classes in order to equal the number of vehicular environments to identify.

Because the previously mentioned state-of-the-art architectures are designed to receive two-dimensional (2D) inputs for shape size, we considered a 2D channel matrix in the input features instead of a 1D channel vector. Thus, we rearranged our dataset from 1D to 2D, as follows:

(8) $$\begin{array}{c} \underset{\left[n*n\right]}{H_{2D}}=Diag\left(\underset{\left[1*n\right]}{H_{1D}}\right) \end{array}$$

where Diag() is the diagonal matrix, $H_{2D}$ and $H_{1D}$ are the channel matrix and corresponding channel vector, respectively, where their coefficients are the CSI values, and *n* is equal to 128, which is equivalent to the number of CSI values estimated per packet; thus, we have an input shape size of 128×128.

In Table 7, our proposed model is compared to the indicated state-of-the-art architectures in terms of average test accuracy (Acc), individual test accuracy for each environment, and the average time required to identify the environment (prediction time).

From Table 7, it is clear that our proposed model has the best performance in terms of prediction time compared to all the other architectures presented in the table, with a prediction time of 39.56μs. The architectures ResNet50, Xception, and InceptionV3 have prediction times around of 700 μs, whereas the InceptionResNetV2, DenseNet201, MobileNetV2, and DCNN [37] architectures have 1621 μs, 1349 μs, 318 μs and 125 μs, respectively. Thus, the prediction time achieved by our model is significantly (at least three-fold) lower than the prediction time attained by the other architectures.

**Figure 12:**
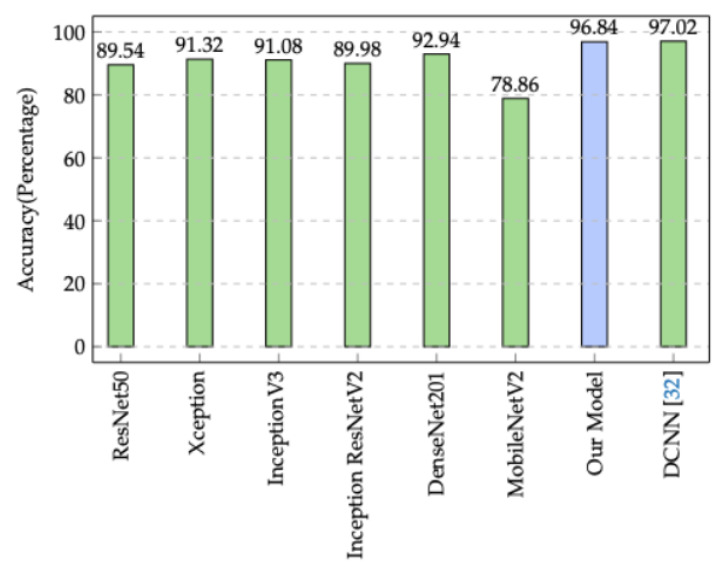
Comparison of our model’s accuracy to state-of-the-art alternatives.

Regarding the overall test accuracy (Figure 12), our model reaches 96.48%. This achieved accuracy is greater than the test accuracy attained by ResNet50, Xception, InceptionV3, InceptionResNetV2, DenseNet201, and MobileNetV2, which have 89.54%, 91.32%, 91.08%, 89.98%, 92.94%, and 78.86%, respectively. Although the proposed DCNN architecture in [37] scores slightly higher than our model in term of test accuracy (by 0.54%), the prediction time achieved by our model is three times faster than DCNN [37].

To attain more insight into the classification performance of the considered architectures, the individual test accuracy of each vehicular environment is presented in Table 7. It can be seen that all the architectures are able to successfully discriminate the H-NLOS environment with accuracy of more than 96%; our model has the best accuracy, at 99.9%. Concerning the H-LOS environment, the individual accuracies are acceptable (close to 80%) for MobileNetV2 and InceptionV3, and the other architectures provide good individual accuracies of around 90%. The test accuracies for the R-LOS environment are less significant compared to other environments. This is due to the channel characteristics of this environment, which are close to the channel characteristics of the U-LOS and H-LOS environments. However, the accuracy for the R-LOS environment is considered good for our model, MobileNetV2, and DCNN (up to 90%), and is acceptable for the other models (around 80%). For the U-LOS environment, the test accuracy is generally around 90%, while our proposed model has the best accuracy at 97.4%. For the U-NLOS environment, all the architectures except MobileNetV2 are able to recognize this environment with high accuracy (up to 93%).

### 5.4. Minimum Performance Overhead and Reliability

Because our proposed vehicular environment identification approach based on CSI is intended to be implemented in autonomous connected cars for use in a time-critical setting, it is important that the environment identification prediction time consistently meet latency requirements in every scenario. According to [38], this hard time limit typically falls within a few milliseconds. As seen in the results, our proposed CSI-based vehicular environment identification model demonstrates a prediction time that is consistently within the **microsecond** range, which is well under the required range time.

In addition, our proposed CNN architecture using the CSI values as an inputs features proves that it can reliably identify different vehicular environments with high accuracy (96.48%) that meets the requirements for autonomous connected cars.

## 6. Conclusions

In this paper, we have presented a deep learning-based vehicular environment identification approach using vehicular wireless channel characteristics in the form of estimated CSI as input features for a CNN model. The results of our validation tests have demonstrate that our methodology can reliably recognize the surrounding environment with high accuracy (96.48%). These same results show that our approach has minimal performance overhead, measured in microseconds, which is well within the expected operational range across various autonomous driving scenarios. In addition, our CNN model has comparable performances to existing state-of-the-art architectures. In summary, our CSI-based vehicular environment identification approach is validated as a reliable solution to enable speed limit decisions for autonomous vehicles. However, because our vehicular environment identification method is based on the use of channel characteristics, it requires a continual exchange of messages. Moreover, we demonstrate promising results with our simulation scenarios. Hence, real-world CSI-based vehicular environment identification testbeds on a larger scale involving more vehicles in specific road conditions remains an open research problem worth investigating in the future. 

## Figures and Tables

**Table 1:** Vehicular environment characteristics.

	Taps	Power [dB]	Delay [ns]	Doppler [Hz]
U-LOS	**Tap 1**	0	0	0
	**Tap 2**	−8	117	236
	**Tap 3**	−10	183	−157
	**Tap 4**	−15	333	492
U-NLOS	**Tap 1**	0	0	0
	**Tap 2**	−3	267	295
	**Tap 3**	−4	400	−98
	**Tap 4**	−10	533	591
R-LOS	**Tap 1**	0	0	0
	**Tap 2**	−14	83	492
	**Tap 3**	−17	183	−295
H-LOS	**Tap 1**	0	0	0
	**Tap 2**	−10	100	689
	**Tap 3**	−15	167	−492
	**Tap 4**	−20	500	886
H-NLOS	**Tap 1**	0	0	0
	**Tap 2**	−2	200	689
	**Tap 3**	−5	433	−492
	**Tap 4**	−7	700	886

**Table 2:** Vehicular environment labels and required speed limits.

Vehicular Environment	Label	Speed Limits
Highway NLOS	0	130 km/h
Highway LOS	1	130 km/h
Rural LOS	2	90 km/h
Urban LOS	3	50 km/h
Urban NLOS	4	50 km/h

**Table 3:** LTS approach accuracy for magnitude angle and two-channel configurations.

Configuration	Accuracy
Magnitude	92.22%
Angle	91.78%
2-Channel	93.42%

**Table 4:** Classification accuracy and average prediction time comparison for LTS approach.

Approach	Accuracy (%)	Prediction Time (μs)
Proposed CNN	93.42	51.33
ANN	86.16	23.11
RF	68.34	25.71
K-NN	63.18	7180
GBN	20.62	4.11
SVM	31.38	10499

**Table 5:** CSI approach accuracy for magnitude angle and two-channel configurations.

Configuration	Accuracy
Magnitude	90.63%
Angle	91.50%
2-Channel	96.48%

**Table 6:** Classification accuracy and average prediction time comparison for CSI approach.

Approach	Accuracy (%)	Prediction Time (μs)
Proposed CNN	96.48	39.56
ANN	85.64	21.11
RF	67.77	24.04
K-NN	59.26	8999
GNB	27.06	4.38
SVM	32.33	15756

**Table 7:** Comparison between our model and state-of-the-art alternatives.

Architecture	H-NLOS Acc (%)	H-LOS Acc (%)	R-LOS Acc (%)	U-LOS Acc (%)	U-NLOS Acc (%)	Acc (%)	Prediction Time (μs)
Our Model	99.9	95.2	92.7	97.4	97.2	96.48	39.56
ResNet50	98.1	88.2	77.8	90.1	93.5	89.54	672
Xception	97.8	91.7	81.4	91.2	94.5	91.32	794
InceptionV3	99.1	79.8	86.9	96.1	93.9	91.08	683
Inception ResNetV2	98.5	89.1	80	86.5	95.8	89.98	1621
DenseNet201	98.5	92.7	85.7	91.2	96.6	92.94	1349
MobileNetV2	96.8	77.8	96	58.2	65.5	78.86	318
DCNN [37]	98.9	96.9	94.3	95.8	99.2	97.02	125

## Data Availability

Not applicable.
